# Grain Products: Traditional and Innovative Technologies

**DOI:** 10.3390/foods13071126

**Published:** 2024-04-08

**Authors:** Manuel Gómez, Eliana Pereira

**Affiliations:** 1 Food Technology Area, Department of Agroforestry Engineering, University of Valladolid, 50 Avda. Madrid, 34071 Palencia, Spain; 2 Mountain Research Center (CIMO), Polytechnic Institute of Bragança, Campus de Santa Apolónia, 5300-253 Bragança, Portugal; eliana@ipb.pt

The current knowledge regarding various cereals, their composition, and their processing methods is extensive. There is a solid understanding of the three most well-known cereals (wheat, maize, and rice), as well as for widely consumed products like bread, pasta, or beer. However, when dealing with other cereals or grains, or with less popular products, the knowledge base is not as robust, necessitating further research to establish a foundational understanding. While the characteristics required for high-quality wheat flour for breadmaking or other purposes are well understood, and in-depth knowledge has been obtained regarding the major commercial varieties and their suitability for different processes [1], the same level of understanding is lacking for other cereals, especially those used in local products or gluten-free alternatives to products traditionally made with wheat. Actions aimed at improving the quality of products intended for special groups, or the nutritional improvement of grain-based products, are also of interest.

This Special Issue delves into the study of various maize genotypes’ influence on polenta production—a staple in certain regions of Italy—and its spread to areas historically influenced by Italian immigration (article 1). Additionally, the Issue addresses the study of Mahewu, a maize-based product that is typical in South Africa, focusing not only on the maize type but also on the inoculation process due to its fermentation (article 2). Furthermore, an article on Bourbon production, a maize-based distilled product, is included (article 3). These studies not only expand the knowledge of these local or less-explored means of production but also lay the groundwork for future research, such as production methodologies or analyses.

Improving gluten-free products, and determining gluten content, remains a highly researched topic. Research in recent decades has transferred to industrial practice, and has significantly enhanced the quality, consistency, and affordability of these products [2,3]. This interest on the part of the industry has also been fueled by the growing number of people who demand gluten-free products, and thus the larger potential market. This is also an aspect that needs to be further investigated, although it is not included in the subject matter of this Special Issue. While gluten-free products generally have lower nutritional quality than wheat-based ones, the consumer base for such products has grown beyond those with celiac disease or wheat allergies. Factors such as FODMAPs [4] or even a potential nocebo effect [5] require continued study.

This Special Issue also addresses pasta production using gluten-free flours like maize or red lentils (articles 4 and 5), as well as the use of galactomannan to enhance the quality of gluten-free bread (article 6). Additionally, it discusses gluten measurement in hydrolyzed forms, such as in beers, where traditional gluten analysis methods are ineffective (article 7). These articles contribute to improvements in gluten-free product quality and enhance the quality of life of those with celiac disease.

The nutritional enhancement of cereal-based products remains a significant area of interest. This is achievable through the incorporation of nutritionally beneficial ingredients or elimination of/reduction in less nutritious ones like simple sugars or saturated fats in items such as cakes or cookies. Research in this area has been extensive. Nutritional improvements can also be achieved through process modification, particularly fermentation conditions in products like bread. Sprouting grains offer another avenue for nutritional enhancement, as evidenced by various studies [6]. Obviously, these changes will depend on the germination time and of germination conditions, and, in addition to modifying the nutritional characteristics of the grains, the functionality of the obtained flours will also be modified. While research on sprouted grains, especially wheat and barley, is extensive [7], further investigation is needed for pseudocereals and legumes.

This Special Issue addresses the germination processes for less-studied cereals like sorghum (article 8) and pseudocereals like buckwheat and quinoa (articles 9 and 10), which are all gluten-free grains that are suitable for gluten-free product development.

Reducing food waste is another pressing issue, in which grain processing plays a crucial role. Fruit and vegetable byproducts can be transformed into flour for use in pasta [8], bread, or other baked goods [9]. Byproducts from cereal-based product manufacturing that are rich in fiber and protein hold significant nutritional value for inclusion in other grain-based or alternative formulations. Byproducts like brewer’s spent grains [10] or okara [11] have received considerable attention in scientific publications. Additionally, repurposing discarded bread has garnered attention in recent years [12]. Continued study and product development aimed at reducing food waste will remain relevant, considering that one-third of the world’s food production goes to waste.
